# Intelligence, Correspondence, &c.

**Published:** 1833-04-01

**Authors:** 


### INTELLIGENCE, CORRESPONDENCE, &c.

### COOKE'S TRANSLATION OF MORGAGNI.

We observe that some copies of that inestimable and inexhaustible treasure of information, the work of Morgagni on the "Seats and Causes of Diseases," translated by Mr. Cooke, are to be procured from any of the London Booksellers for the trifling sum of Sixteen Shillings! We recommend those of our readers who do not possess the work, to order it immediately.

Dr. Gilchrist's paper on Homeopathy has been received.

Dr. Donovan's case and drawing of Disease of the Tongue, came safe to hand.

Dr. O'Beirne's work in our next.

Our article "Excerpta Choleralogica" is deferred till next quarter, which will account to several correspondents for omissions in this number.

A translation of Mad. Boivin's Maladies de l'Uterus, by Mr. Hemming, is in the press.

